# Self-Management Assessment Instruments for Adults with Colorectal Cancer-Related Intestinal Stomas: A Scoping Review

**DOI:** 10.3390/healthcare14172762

**Published:** 2026-09-01

**Authors:** Juanfang Zhang, Yuanyu Liao, Sisi Zhang, Xiaomei Wei, Hailan Peng, Qiong He, Danfeng Li, Huan Wang

**Affiliations:** 1 Department of Nursing, Union Hospital, Tongji Medical College, Huazhong University of Science and Technology, 1277 Jiefang Avenue, Wuhan 430022, China; 2015xh1176@hust.edu.cn (J.Z.); 2014xh1063@hust.edu.cn (S.Z.); 2005xh0939@hust.edu.cn (X.W.); 2004xh0991@hust.edu.cn (H.P.); 2009xh1059@hust.edu.cn (Q.H.); 2014xh1226@hust.edu.cn (D.L.); 2 Department of Gastroenterology, Shenzhen Hospital, Southern Medical University, Shenzhen 518000, China; 2012xh0819@hust.edu.cn

**Keywords:** intestinal stomas, self-management, assessment instruments, psychometric properties, scoping review

## Abstract

**Highlights:**

**What are the main findings?**

- This scoping review systematically mapped 12 self-management assessment instruments across 32 studies focusing on adults with colorectal cancer-related intestinal stomas.
- All included instruments reported Cronbach’s α values ranging from 0.805 to 0.977, yet most lacked evidence of structural validity, test–retest reliability, measurement error, and responsiveness.
- Four core self-management domains were identified: stoma technical care, symptom and complication management, psychological adaptation, and family-social interaction. Only three of the 12 instruments were developed under explicit theoretical frameworks.
- Multiple adapted versions of the same instrument (e.g., original ESCA and its Chinese revision) are widely used without formal measurement invariance testing.

**What are the implications of the main findings?**

- The available literature does not support comprehensive evaluation of the psychometric robustness of existing ostomy self-management tools.
- Inconsistent equivalence testing across scale versions hinders cross-study comparison and limits generalizability of findings.
- Future research should prioritize cross-cultural adaptation, supplementary psychometric testing of unreported properties, and development of brief simplified tools. New scale development is only warranted when current instruments fail to capture patients’ core rehabilitation demands.

**Abstract:**

**Background/Objectives:** Self-management for adults living with intestinal stomas secondary to colorectal cancer encompasses technical ostomy care, physical symptom control, psychological adjustment, and social participation, creating demand for standardised assessment instruments to identify unmet rehabilitation needs. Definitions of generic self-care and stoma-specific self-management remain inconsistent across published literature, and psychometric indicators are incompletely reported across available measurement instruments. This review includes both stoma-specific instruments and generic self-care scales that have been validated or widely applied specifically in colorectal cancer ostomy cohorts. Following JBI scoping review methodology, this work systematically maps all published self-management assessment instruments developed for adults with intestinal stomas secondary to colorectal cancer, summarises their core measurement domains and documented psychometric properties, and identifies prevailing gaps in scale development and validation research. **Methods:** This scoping review was conducted in accordance with Joanna Briggs Institute methodological guidance and the Preferred Reporting Items for Systematic Reviews and Meta-Analyses Extension for Scoping Reviews (PRISMA-ScR) reporting checklist. Ten electronic databases were searched from inception to 31 December 2025, with supplementary manual screening of reference lists. Eligible sources were peer-reviewed English or Chinese articles focusing exclusively on adults with colorectal cancer–related intestinal stomas. Two independent researchers completed title/abstract and full-text screening, followed by data extraction using a piloted standardised form. Consistent with descriptive scoping review design, no formal methodological quality evaluation or risk-of-bias assessment was performed. **Results:** Thirty-two studies involving 12 self-management assessment instruments were finally included. All tools documented Cronbach’s α coefficients ranging from 0.805 to 0.977. Content validity indicators were documented for eight instruments, whereas construct validity verified via factor analysis or structural equation modelling was only available for three tools. Criterion-related validity, cross-cultural validity and responsiveness were scarcely reported across most instruments. Only three instruments were fully developed based on complete and clearly articulated theoretical frameworks. Four consistent core measurement domains were identified across scales: stoma technical care, symptom and complication management, psychological adaptation, and family-social interaction. **Conclusions:** Although internal consistency metrics are universally reported, comprehensive multi-faceted validity testing and responsiveness evaluation are largely absent from existing published evidence. Cross-cultural measurement invariance between original and locally adapted versions (such as the ESCA and its Chinese revised forms) has rarely been examined. Future research priorities include rigorous cross-cultural adaptation following International Society for Pharmacoeconomics and Outcomes Research (ISPOR) and Consensus-based Standards for the selection of health Measurement INstruments (COSMIN) protocols, full psychometric verification of currently available instruments, and development of brief validated short forms for clinical screening. New instrument development should only be initiated if existing tools fail to capture core rehabilitation priorities identified by patients, caregivers, and clinical staff. No responsiveness metrics were identified across the 32 included colorectal cancer-specific clinical studies. The absence of relevant evidence in this literature pool does not prove that these instruments cannot detect longitudinal changes; formal responsiveness testing is required before applying these tools for long-term outcome monitoring.

## 1. Introduction

In 2022, the International Agency for Research on Cancer (IARC) reported that colorectal cancer accounted for approximately 1.93 million new cases and 904,000 deaths worldwide, ranking it the third most frequently diagnosed malignancy and the second leading cause of cancer mortality globally [1]. Approximately 50–60% of patients diagnosed with colorectal cancer undergo intestinal stoma construction. Globally, more than one million individuals live with a colorectal cancer-related stoma, and approximately 100,000 new cases are identified each year [2,3].

Long-term care for adults with colorectal cancer-associated stomas creates considerable financial strain, covering expenses for stoma appliances, complication management, and nutritional support [4,5,6]. A cohort study conducted in Jiangsu Province of China reported that 75.3% of families experienced financial stress related to routine stoma care [4], while a Danish study found that stoma-related hospitalizations increased overall inpatient expenditures by 8–16% [5]. Beyond economic strain, many adults with colorectal cancer-related intestinal stomas face psychological distress. Approximately 30–50% develop clinically significant anxiety or depression, with consistent evidence linking altered body image to social withdrawal [7].

Beyond these burdens, effective self-management directly accelerates postoperative recovery and improves long-term quality of life. The World Health Organization (WHO) identifies self-management as a core component of chronic disease care, enabling patients to take an active role in managing their health and reducing reliance on continuous professional support [8]. Most hospital-based training programs focus on hands-on technical stoma care skills, while few standardized assessment tools are available for post-discharge home care. Consequently, many adults with colorectal cancer-related intestinal stomas present to emergency services after discharge because they cannot identify early warning signs of complication or perform basic stoma care correctly, which delays recovery and increases healthcare service utilization [9].

A vital conceptual distinction must be clarified before reviewing existing assessment tools: generic self-care agency—as measured by instruments such as the ESCA—refers to universal daily self-care capacity applicable across chronic disease populations, whereas ostomy-specific self-management addresses unique disease demands including stoma maintenance, peristomal complication monitoring, dietary adjustment, and psychological adaptation. These constructs are not interchangeable and require separate measurement frameworks. Notably, multiple adapted ESCA versions are retained in this review because they have been widely validated and exclusively applied to colorectal cancer-related stoma cohorts within our included studies; generic self-care scales without ostomy population application records are excluded per our criteria.

Effective ostomy-specific self-management accelerates postoperative recovery, reduces complication risk, lowers medical costs, and improves quality of life [2]. However, self-management competence varies considerably among discharged colorectal cancer ostomy patients, and many exhibit suboptimal self-management proficiency. Moderate self-management capacity has been reported in patients with colorectal cancer-related intestinal stomas [10], with limited opportunities to practice independent stoma care during hospitalization [11]. Additionally, patients consistently seek education on stoma maintenance, dietary adjustment, and complication recognition to address the physical and psychosocial challenges after surgery [12].

A preliminary search of MEDLINE, the Cochrane Database of Systematic Reviews, and JBI Evidence Synthesis was undertaken prior to protocol finalisation. No completed or ongoing scoping reviews or systematic reviews focusing specifically on self-management assessment instruments for adults with colorectal cancer-related intestinal stomas were identified.

A scoping review methodology was chosen over a systematic review approach because the review aims to map the breadth of available instruments, classify diverse methodological papers, and identify knowledge gaps rather than to calculate pooled quantitative effect sizes or conduct formal risk-of-bias assessments of intervention studies [13].

Improving self-management capacity in adults with colorectal cancer-related intestinal stomas depends on psychometrically sound measurement instruments. Accurate assessment provides the foundation for identifying patients’ self-management levels and for designing tailored nursing interventions. A range of self-management scales have been developed for this specific patient group, including the Enterostomy Patient Self-Management Questionnaire (EPSMQ), the Exercise of Self-Care Agency Scale (ESCA), the Specific Self-Care for Ostomized Patients Questionnaire (CAESPO), and the Ostomy Self-Care Index (OSCI). However, these instruments vary substantially in their dimensional structures, target populations, and reported psychometric evidence. Several scales lack comprehensive validity testing, and cross-cultural validation remains insufficient for instruments adapted across different clinical contexts [14]. The extent of these variations and their implications for clinical practice have not been systematically examined.

Three core research gaps are evident in the current literature. First, definitions of generic self-care and stoma-specific self-management are inconsistently delineated, with many instruments conflating generic self-care agency with the unique disease-specific behavioural demands of ostomy self-management. Second, the dimensional structures and psychometric properties of available instruments are incompletely reported, particularly for scales developed in Chinese populations, where comprehensive validity metrics apart from internal consistency coefficients are often limited. Third, multiple adapted versions of the same original instruments are widely used without formal measurement invariance testing, limiting meaningful comparison across studies and populations.

To address these gaps, this scoping review systematically maps all available self-management assessment instruments developed for or applied to adults with colorectal cancer-related intestinal stomas, and it summarizes their structural characteristics, reported psychometric properties, and evidence gaps in cross-cultural adaptation research.

## 2. Materials and Methods

This scoping review was pre-planned prior to any database retrieval. The first round of primary database searches was conducted on 15 August 2025, followed by initial title, abstract, and full-text screening. The complete formal review protocol was registered on the Open Science Framework (OSF) on 22 January 2026 (registration DOI: 10.17605/OSF.IO/WMJSF). Supplementary instrument-targeted searches across all databases were completed on 31 July 2026 after protocol registration. All deviations between the pre-registered OSF protocol and the final manuscript are fully documented in Supplementary S1, and the full original OSF registration document is uploaded as an independent supplementary file per journal requirements. This review strictly followed the latest JBI scoping review methodological guidances [13] and complied with the PRISMA-ScR [15] reporting checklist. Ethical approval was not required, as this study only synthesizes published secondary literature without collecting original data from human participants.

### 2.1. Review Questions

Three research questions guided this review: (1) What structural characteristics, dimensional frameworks, content domains, and scoring protocols are reported for existing self-management assessment instruments developed for adults with colorectal cancer-related intestinal stomas? (2) What psychometric properties have been reported for these instruments in the included studies? (3) In what clinical and research contexts have these instruments been applied?

All discrepancies between the pre-registered OSF protocol and the final manuscript—including the addition of the CINAHL and Scopus databases and supplementary instrument-targeted searches—are fully documented in the revised Supplementary S1.

### 2.2. Inclusion and Exclusion Criteria

Screening criteria were formulated following the PCC (Population, Concept, Context) framework to standardize study selection, with each dimension mapped to corresponding inclusion rules below. Studies were eligible for inclusion only if they met all of the following criteria:

(1) Population: Adult participants aged ≥18 years with histologically confirmed colorectal cancer and a history of colostomy or ileostomy construction;
(2) Concept: Original research focusing on the development, cross-cultural adaptation, validation, or clinical application of self-management measurement instruments;
(3) Context: Data collection conducted within hospital inpatient, community outpatient, or home rehabilitation settings.

Records were excluded based on any of the following conditions:

(1) Full texts could not be retrieved after contacting authors and interlibrary loan requests;
(2) Duplicate publications reporting identical scale datasets from the same research group;
(3) Non-original research: conference abstracts, narrative/systematic reviews, meta-analyses, book chapters, commentary articles;
(4) Participants with intestinal stomas resulting from inflammatory bowel disease, abdominal trauma, congenital gastrointestinal disease, or other non-oncological disorders;
(5) Generic chronic disease self-care scales without targeted adaptation for adults with colorectal cancer-related intestinal stomas.

Two categories of measurement instruments satisfy the eligibility criteria of this review: (1) scales originally developed or culturally adapted exclusively for adults with colorectal cancer-related intestinal stomas; (2) generic self-care inventories such as the ESCA series that have been widely validated and applied within colorectal cancer ostomy cohorts without disease-specific revision. All original and localized modified versions of ESCA are listed separately in Table A2 to avoid conflation of distinct scale variants.

Qualitative studies, conference abstracts, standalone study protocols without empirical data, and unpublished grey literature were excluded. Only peer-reviewed full-text original articles published in English or Chinese were retained.

### 2.3. Search Strategy

The literature search was divided into three chronological phases with clear boundaries.

**Figure 1:**
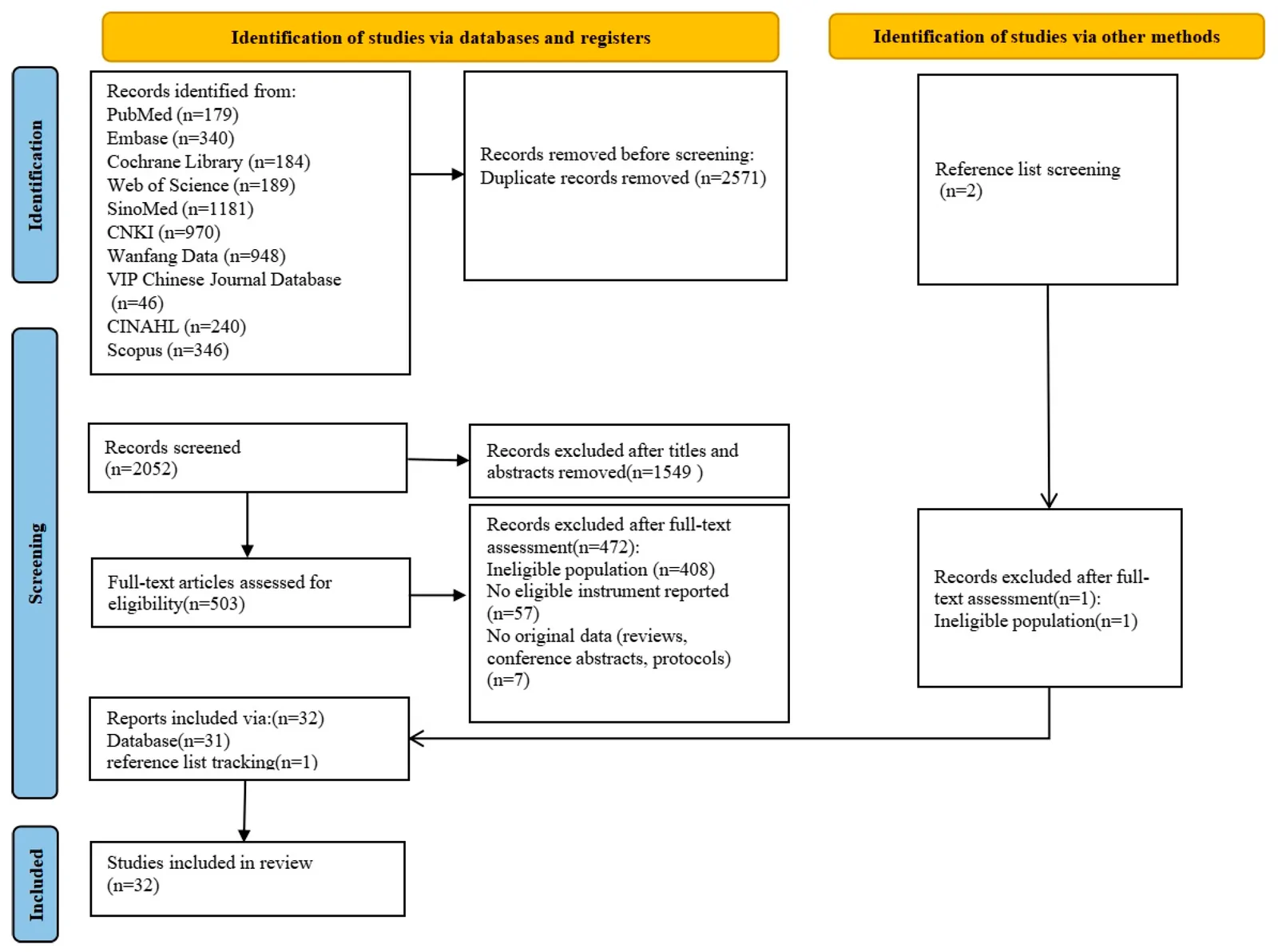
PRISMA flow diagram.

The preliminary eight-database search was performed on 15 August 2025 across eight core databases; the search strings contained and returned 8939 low-relevance citations. This entire retrieval dataset was fully discarded, and the search strategy was comprehensively revised. After deleting redundant search terms, we expanded the retrieval scope to ten databases (adding CINAHL and Scopus) and re-ran the full literature search from scratch, yielding 4623 raw records before deduplication; This unified record count aligns with the data presented in Figure 1 and Supplementary S1.

The complete review protocol was prospectively registered on OSF on 22 January 2026 (DOI: 10.17605/OSF.IO/WMJSF). Supplementary instrument-targeted searches were conducted on 31 July 2026 across all ten databases, using full scale names, standard abbreviations and original developer information to retrieve missing scale development and validation studies. All literature retrievals were limited to publications released from database inception to 31 December 2025. Additional manual screening of reference lists from all included papers was performed to capture overlooked relevant studies without generating duplicate primary records. All deviations between the pre-registered OSF protocol and the final search workflow are fully documented in Supplementary S1.

After the primary database retrieval, supplementary targeted searches were performed for each unique instrument identified during preliminary screening, using full scale names, standard abbreviations and original developers to capture missing scale development and validation papers.

### 2.4. Literature Screening and Data Extraction

All search results were exported to NoteExpress V4, and duplicate entries were removed via automated tools followed by manual cross-checks. The complete screening process is summarised in the PRISMA-ScR flow diagram (Figure 1). Two trained researchers (J.Z. and Y.L.) independently screened titles, abstracts, and full texts against the predefined eligibility criteria. Disagreements were resolved through joint discussion, with a third senior reviewer (H.W.) providing final adjudication when consensus could not be reached. Cohen’s Kappa for full-text screening was 0.85, indicating strong inter-reviewer agreement. For manuscripts whose full texts could not be initially retrieved, corresponding authors were contacted twice by email, and interlibrary loan services were utilised to obtain complete documents where possible.

A standardized data extraction form was developed based on JBI scoping review guidance and COSMIN terminology, and pilot-tested on five randomly selected eligible included studies before formal data extraction. Two trained researchers (J.Z. and Y.L.) independently extracted data from all included studies using the finalized template. Disagreements during data extraction were resolved through discussion; if consensus could not be reached, a third senior reviewer (H.W.) provided final adjudication. All psychometric coefficients and instrument characteristics were extracted exactly as reported in the original publications; no secondary recalculation or reanalysis of raw data was performed.

Extracted variables fell into three categories:

(1) Study metadata: authors, publication year, study country, research design, sample size, and clinical setting; (2) Instrument characteristics: full scale name, original developer, source language, theoretical framework, total number of items, subscale dimensions, and scoring protocol; and (3) Measurement properties reported in primary studies: reliability (internal consistency, test–retest reliability); validity (content validity, construct validity, criterion validity, and cross-cultural validity); and responsiveness.

Clarification on ESCA inclusion: While the original ESCA is a generic self-care scale, its multiple Chinese revised iterations were repeatedly validated and solely administered to adults with colorectal cancer-related intestinal stomas across our 32 included studies, thus satisfying our eligibility rule for mapped instruments. Each distinct ESCA version is listed separately in Table A2 to display divergent psychometric results, yet ESCA is counted as a single core instrument in quantitative statistics. Pure generic self-care scales without documented application in colorectal cancer-related intestinal stoma cohorts are excluded per our predefined exclusion criteria.

When multiple publications reported data for a single instrument, information from the original scale development study was prioritised. For instruments with distinct revised, translated, or culturally adapted iterations (e.g., original ESCA and its Chinese revised version), each variant was documented separately and not treated as equivalent unless formal measurement invariance was confirmed within the included literature.

### 2.5. Critical Appraisal

Standard analytical workflows adopted in this scoping review are outlined below. Consistent with the core objective of mapping available measurement tools rather than evaluating their methodological quality, no COSMIN-based quality ranking was conducted during data synthesis.

Dual independent dimension coding: Two researchers grouped synonymous subscale labels, and discrepancies were resolved by a senior third reviewer. Only distinct core instruments were counted for frequency statistics; multiple adapted ESCA versions were not double-counted.

Dual-layer evidence stratification: Two separate evidence pools were defined: (1) clinical data from the 32 included colorectal cancer stoma studies; (2) unrestricted original scale development papers to eliminate artificial psychometric gaps caused by strict population eligibility filters. Unreported metrics in included clinical papers do not equate to inherent flaws of the corresponding instruments, as relevant validation evidence may exist in excluded general ostomy literature.

Theoretical data extraction: Only verbatim theoretical descriptions extracted from original scale development articles were recorded in Supplementary S3, and no subjective alignment ratings (high/medium/low) were assigned.

Psychometric coding: Each psychometric indicator was categorised as fully reported, partially reported, or unreported, without subjective overall quality judgements for individual instruments.

## 3. Results

### 3.1. Search Results

A total of 4623 records were initially retrieved through database searches. Two additional records were retrieved through manual screening of reference lists, bringing the total unduplicated pool to 4625 records prior to deduplication. After duplicate removal, title and abstract screening, and full-text assessment, 32 studies were ultimately included in this review [12,16,17,18,19,20,21,22,23,24,25,26,27,28,29,30,31,32,33,34,35,36,37,38,39,40,41,42,43,44,45,46], including 28 Chinese studies and 4 English studies. The entire screening workflow strictly followed the PRISMA-ScR reporting standards (see Figure 1).

### 3.2. Basic Characteristics of Included Studies

The 32 eligible primary studies originated from four countries: China (n = 28), Italy (n = 1), Spain (n = 2), and Portugal (n = 1), while the 12 measurement instruments were originally developed across five countries (China, the United States, Italy, Spain, and Portugal). All papers were published between 2013 and 2025. Regarding research design, there were 18 single-center and 14 multi-center investigations. The study types comprised 17 cross-sectional works, 3 longitudinal trials, 8 randomized controlled trials, 2 quasi-experimental studies, 1 pilot study, and 1 methodological study. Based on core research objectives, the papers were divided into four subgroups: 24 studies adopted validated self-management scales to assess adults with colorectal cancer-related intestinal stomas; 4 studies developed original measurement tools alongside clinical validation; 3 focused on full psychometric verification of newly designed instruments; and 1 solely adopted methodological analysis of scale construction. Consistent with the PCC framework specified in JBI scoping review guidance, all studies were categorized by data collection settings. Sixteen studies (50.0% of all 32 included studies) collected inpatient perioperative data, fifteen (46.9%) adopted community-only follow-up designs, and one mixed-setting study accounted for 3.1% of all 32 studies. All percentages were calculated based on the full sample of 32 primary studies as the denominator. Among all 32 eligible papers, 31 reported reliability indicators and 21 documented complete validity-related psychometric evidence, indicating that internal consistency data are more consistently reported than validity indicators across the included literature. Full descriptive details of each primary study are summarised in Table A1.

### 3.3. Characteristics of Included Assessment Tools

**Figure 2:**
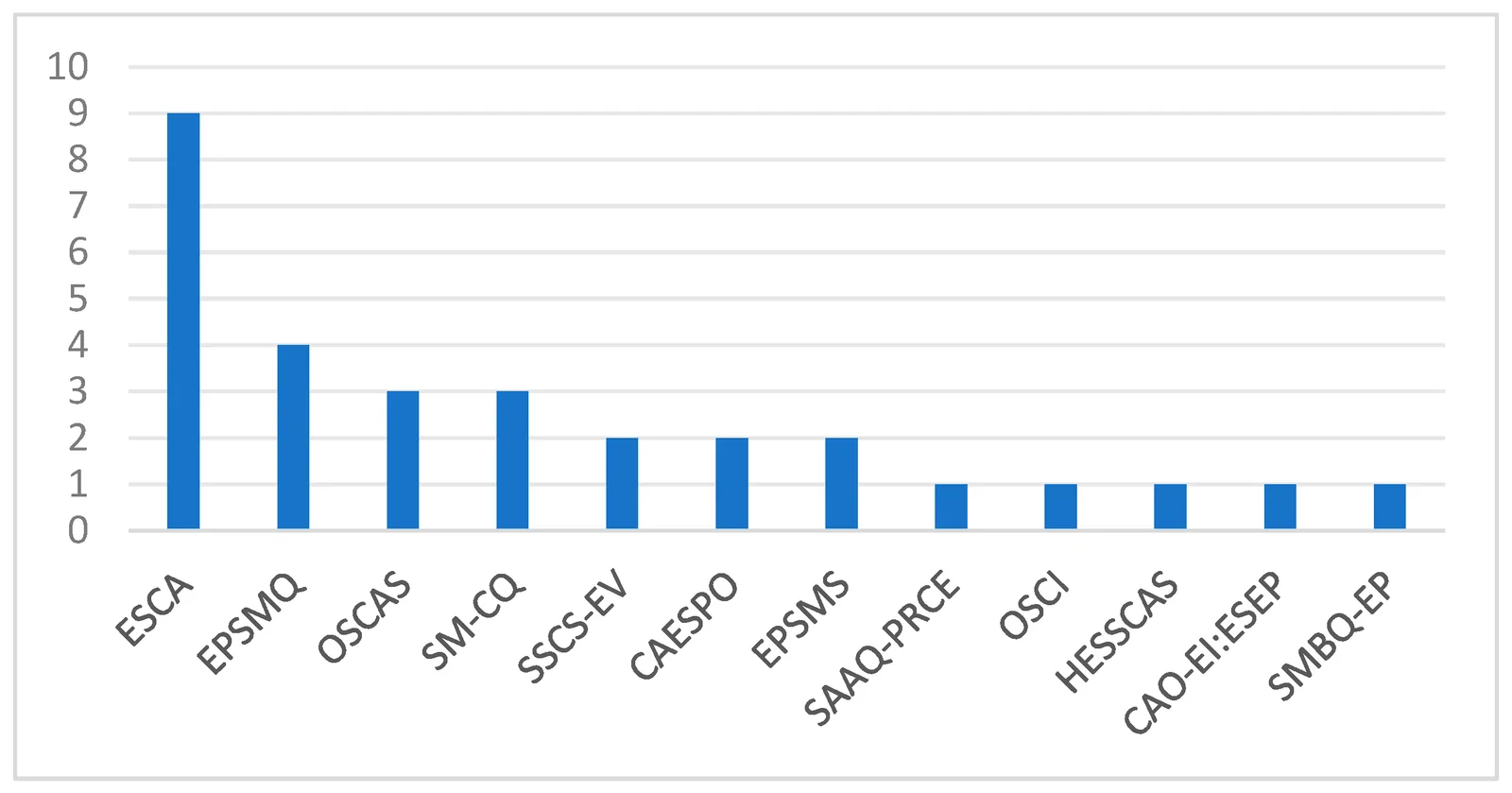
Citation frequency of each included self-management instrument. Frequency counts represent the number of independent primary studies (n = 32) that adopted the corresponding scale for participant assessment.

12 self-management assessment tools targeting adults with colorectal cancer-related intestinal stomas were identified. These instruments were originally developed by research teams across five countries: China, the United States, Italy, Spain, and Portugal. The ESCA was the most frequently adopted tool, cited in nine included studies. Several culturally adapted Chinese versions of the ESCA have been published with divergent psychometric properties; all original and localized ESCA iterations are tabulated separately in Table A2 to avoid conflation of distinct versions (Figure 2).

Of note, the Ostomy Self-Care Index (OSCI) is available in two distinct versions: the patient self-care version (OSCI) and the caregiver contribution version (CC-OSCI). These two instruments measure different constructs and are analysed separately in Table A2.

### 3.4. Overview of Theoretical Foundations, Reported Psychometric Properties, and Cross-Cultural Adaptation Status

#### 3.4.1. Theoretical Foundations of Included Instruments

Of the 12 included instruments, three were developed based on clearly articulated theoretical frameworks: the ESCA (Orem’s self-care deficit theory), the HESSCAS (Knowledge–Attitude–Practice model combined with self-efficacy theory), and the OSCI (Riegel’s Middle-Range Theory of Self-Care of Chronic Illness). The OSCI‘s caregiver counterpart (CC-OSCI) shares the same theoretical foundation and is listed separately in Table A2 to distinguish the patient version from the caregiver version, without being counted as an independent instrument. Three additional instruments (EPSMQ, SMBQ-EP, and SM-CQ) drew on partial core concepts from established models, yet empirical evidence verifying the matching relationship between theoretical constructs and scale dimensions remained inconsistent. The remaining six instruments were derived predominantly from clinical expertise and literature reviews rather than from a priori theoretical frameworks; even where theoretical models were superficially cited, researchers failed to illustrate how these theories systematically guided item design. Their dimensional structures were inductively derived from clinical observation and expert opinion, rather than deductively from formal theoretical systems. Supplementary S3 records the theoretical sources of instruments with clearly articulated theoretical frameworks, without assigning subjective high/medium/low consistency ratings.

#### 3.4.2. Distribution of Reported Psychometric Evidence

Internal consistency (Cronbach‘s α) was reported for all 12 instruments, with values ranging from 0.805 to 0.977. Among validity assessments, content validity was most frequently documented (n = 8), whereas construct validity—examined via factor analysis or structural equation modeling—was available for only three instruments (SM-CQ, CAESPO, and SMBQ-EP). Criterion-related validity was barely documented across any instrument. Evidence for cross-cultural validity and responsiveness was not reported in the included studies.

It is critical to clarify that incomplete psychometric data in the 32 included clinical studies targeting adults with colorectal cancer-related intestinal stomas do not equate to inherent defects of these assessment tools. Relevant validation evidence may exist in broader ostomy population research excluded by the strict eligibility criteria of this review; the observed pattern merely reflects the scope of our included studies, rather than fundamental shortcomings of the measurement instruments. Full reliability and validity data for each instrument are summarized in Table A2.

#### 3.4.3. Cross-Cultural Adaptation and Applicability Considerations

The 12 included instruments were originally developed in five countries: China, the United States, Italy, Spain, and Portugal). Direct application of instruments developed in one cultural context to populations in another setting without formal validation may introduce measurement bias, as item interpretation and response patterns can be influenced by cultural norms, health beliefs, and caregiving practices. Rigorous cross-cultural adaptation following the ISPOR and COSMIN guidelines is essential before instruments can be confidently used across different cultural contexts. This process should include: (1) forward-backward translation; (2) multidisciplinary expert review; (3) cognitive debriefing with target population representatives; and (4) formal measurement invariance testing.

It should be noted that Turkish and Brazilian cross-cultural adaptation studies for OSCI and CC-OSCI have been published in external literature; however, these papers did not meet our colorectal cancer-only population eligibility filter and were therefore not retrieved in this review. Limited cross-cultural data within our included studies does not mean such global validation research is absent.

## 4. Discussion

### 4.1. Summary of Main Findings

This scoping review identified 12 self-management assessment instruments designed or revised for adults living with an intestinal stoma after colorectal cancer surgery, extracted from a total of 32 primary studies conducted across five countries. These instruments show clear divergence in dimensional composition, theoretical underpinnings, and the comprehensiveness of reported psychometric indicators.

Nearly all instruments adopt multi-dimensional structures, yet their measurement priorities are distributed unevenly. Physical stoma care skills—including routine ostomy maintenance, symptom monitoring and complication prevention—occupy the majority of measurement items. By comparison, dimensions covering psychological adjustment, social reintegration, and sustained behavioral modification receive far less elaboration and descriptive detail in most scales. It is hypothesised that this uneven weighting of measurement domains arises from a biomedical orientation embedded in standard ostomy clinical practice, which places greater emphasis on physical ostomy management than holistic psychosocial self-care. Clinical work tends to prioritize hands-on technical guidance and postoperative regimen adherence, while stoma self-management is rarely framed as a holistic recovery process covering physical, psychological, and social wellbeing [47]. If clinicians rely solely on these existing instruments for regular assessment, subtle unmet needs among community-based ostomy survivors, such as persistent emotional distress, social withdrawal, and negative body image, may remain undetected.

The majority of instruments lack robust theoretical support during development, a key observation elaborated in Section 3.4.1. Only three scales were built around complete, well-documented theoretical frameworks that guided item generation and dimension partitioning: the ESCA, grounded in Orem’s self-care deficit theory, and HESSCAS, constructed from a combined Knowledge–Attitude–Practice and self-efficacy model, and OSCI, developed based on Riegel’s Middle-Range Theory of Self-Care of Chronic Illness. Three further instruments (EPSMQ, SMBQ-EP, and SM-CQ) partially drew on established theoretical concepts, without full empirical verification of theory-dimension alignment. The remaining six instruments were developed mainly through clinical experience and literature collation, with dimensions summarized inductively from frontline clinical observations rather than deductively derived from established theories.

Notably, multiple culturally adapted Chinese versions of the ESCA are presented separately in Table A2 to illustrate heterogeneous psychometric findings, while ESCA was counted as a single instrument in quantitative syntheses. The caregiver-oriented CC-OSCI was also listed independently in Table A2 but was not included in the final 12-instrument dataset.

Tools without clear theoretical foundations are still usable in clinical and research settings, but the absence of a unified guiding framework often leads to overlapping subscale boundaries and inconsistent structural logic. This creates challenges when interpreting scale scores and limits the precision of targeted, individualised nursing intervention planning.

### 4.2. Psychometric Evidence: Internal Consistency and Gaps in Validity Reporting

Every one of the 12 included instruments reported internal consistency results, with Cronbach’s α coefficients ranging from 0.805 to 0.977. Such values reflect acceptable within-scale item homogeneity, yet internal consistency alone cannot fully demonstrate a scale’s reliable measurement performance. As Streiner and Norman have noted, extremely high Cronbach’s α values may signal redundant items rather than superior measurement precision, especially when structural validity has not been formally verified [48].

Validity-related evidence is far less comprehensive across the included tools, as summarised in Section 3.4.2. Content validity indicators were documented for eight instruments, while structural validity formally examined via exploratory factor analysis or structural equation modelling was only reported for three tools: SM-CQ, CAESPO, and SMBQ-EP. None of the instruments retrieved in this review presented criterion-related validity data. Formal cross-cultural adaptation procedures were reported for EPSMQ and CAESPO, yet measurement invariance test results were not documented in their original validation publications. No tool contained published responsiveness data, a critical property required for tracking dynamic changes in self-management over long-term follow-up.

It is important to clarify that incomplete reporting of these psychometric metrics within our included 32 studies reflects the research scope and priorities of original validation studies, rather than inherent flaws within the measurement instruments themselves. Relevant psychometric evidence may exist in broader ostomy-related literature that was excluded according to this review’s eligibility criteria.

Our findings align closely with the conclusions put forward by Goodman et al. [2]. Their systematic review of ostomy self-management interventions pointed out that most clinical trials rely on outcome scales supported by incomplete or ambiguously reported psychometric evidence. The present scoping review extends this work by systematically mapping which COSMIN-recommended measurement properties have been formally documented for each instrument. The heavy concentration of internal consistency and content validity data, paired with widespread missing records for construct validity, criterion validity, cross-cultural equivalence, and responsiveness, highlights a prominent evidence gap requiring further targeted psychometric research.

### 4.3. Cross-Cultural Applicability and Considerations for Scale Adaptation

All 12 instruments were originally developed in five distinct nations: China, the United States, Italy, Spain, and Portugal, each with unique healthcare delivery systems, cultural norms and patient expectations around self-management. Instruments validated in one cultural setting cannot be directly applied to other groups without formal cross-cultural revision work.

At present, few empirical studies within the colorectal cancer-specific literature have tested the cross-cultural measurement equivalence of instruments developed outside China when administered to adults with colorectal cancer-related intestinal stomas in China. Direct use of unmodified instruments developed in other cultural contexts without population-specific validation carries a risk of biased assessment results. For future translation and cultural adaptation work, researchers should follow unified protocols released by ISPOR and COSMIN to ensure transparent, replicable methodological procedures.

### 4.4. Comparative Analysis with Previous Published Reviews

Compared with previously published scoping reviews covering general ostomy self-management tools or broad chronic disease self-management scales, this work offers three supplementary analytical perspectives to existing literature:

Strict population restriction: This review exclusively focuses on adults with colorectal cancer-related intestinal stomas, separating this subgroup from patients with stomas caused by IBD, trauma or congenital disease—a distinction rarely adopted in prior mapping studies such as Packer et al. [49] and the 2026 Ostomy Assessment Systematic Integration of Studies (OASIS) scoping review [50].

Dual-language retrieval strategy: Simultaneous searches across English and Chinese databases capture locally developed Chinese ostomy assessment instruments that are largely overlooked in English-only international reviews.

Two-layer evidence differentiation: Separate extraction of clinical application data and original scale validation evidence to reduce artificial psychometric gaps caused by strict population filters, an analytical approach rarely applied in similar scoping reviews.

While Lisboa et al. [51] and the OASIS research team [50] have outlined broad ostomy self-care constructs and available measurement tools, few prior published reviews systematically summarise the full psychometric reporting landscape for colorectal cancer-specific ostomy self-management instruments via bilingual English–Chinese literature retrieval.

### 4.5. Practical Guidance for Clinical Use

Readers should interpret all practice-related guidance presented here with caution, given the incomplete psychometric evidence base across most included instruments. No scale can currently be recommended as superior on the basis of available evidence.

First, scale selection ought to be determined by clear assessment objectives and clinical workflow constraints, rather than habitual routine use. For brief pre-discharge screening on busy inpatient wards, short-form tools with preliminary validity records such as the 10-item SSCS-EV offer practical convenience for identifying gaps in patients’ basic self-management abilities [19]. For sustained community follow-up, multi-dimensional instruments with comprehensive domain coverage including EPSMQ and locally validated SMBQ-EP can capture richer information on overall self-management status [17,33]. In scenarios targeting home safety assessment, complication prevention, and emergency coping capacity evaluation, HESSCAS—built on integrated KAP and self-efficacy theories—contains dedicated home safety dimensions and can be considered for such assessment purposes.

It must be emphasised that these suggestions are drawn purely from item content, domain range and clinical suitability, rather than head-to-head comparative psychometric testing. No published studies have to date formally compared the measurement performance of these instruments, and all tools lack responsiveness data required to track meaningful long-term changes in self-management ability.

Second, clinicians need to interpret scale scores conservatively, bearing in mind the widespread absence of construct validity, criterion validity, and responsiveness evidence. Scale results should support, rather than replace, holistic clinical judgement and comprehensive nursing evaluation. Additionally, nearly all instruments lack validated clinical cut-off values, limiting their utility for patient stratification or clinical diagnostic classification.

Third, every instrument identified in this review relies entirely on patient self-reported data. Self-report methodology is not inherently flawed for measuring subjective constructs such as health knowledge, self-confidence, perceived self-management competence, and psychological adjustment—outcomes that can only be captured through patients’ personal perspectives. Limitations emerge when self-report becomes the sole data source for observable skills that could also be assessed via nurse observation, clinical indicators, or objective medical records. Where clinical workflows permit, combining questionnaire self-report data with objective metrics including peristomal skin condition, complication occurrence, and hospital readmission records can enrich assessment outcomes and mitigate potential response bias.

Fourth, researchers and clinicians planning to implement scales developed outside China within local populations should avoid simple literal translation. Rigorous cross-cultural adaptation workflows—including forward-backward translation, expert content review, cognitive patient interviews, and cross-group measurement invariance testing—are necessary to guarantee cultural fit and stable psychometric performance within the new study population.

### 4.6. Methodological Considerations and Review Limitations

Several methodological boundaries should be acknowledged when interpreting this review’s descriptive findings.

First, consistent with standard JBI scoping review protocols, this study did not conduct formal quality appraisal or risk-of-bias evaluation of original validation studies using the full COSMIN risk-of-bias checklist. The analysis only records which psychometric properties have been reported in published literature, without judging the methodological rigour of validation research or grading the overall certainty of measurement evidence. This descriptive mapping aligns with the core objective of a scoping review: to outline the landscape of available instruments and their documented properties, rather than to rank or judge tool quality.

Second, the literature search was restricted to English and Chinese publications. While this strategy enabled full coverage of domestic Chinese and mainstream international literature, it may exclude validated instruments developed in Spanish, Portuguese, Italian and other non-English, non-Chinese linguistic contexts. This limitation carries particular relevance for our cross-cultural adaptation discussion; future multi-lingual reviews will deliver a more complete global overview of ostomy self-management assessment tools for adults with colorectal cancer-related intestinal stomas.

Third, this review did not independently search PsycINFO or grey literature repositories for unpublished validation data or draft instruments. Nevertheless, the combined search across ten electronic databases (including nursing-focused CINAHL and Scopus), manual reference tracking and secondary targeted searches for each identified scale’s original development paper likely captured the majority of peer-reviewed published validation studies available in English and Chinese.

Fourth, reporting completeness varied widely across the 32 included primary studies. For tools such as OSCI, key psychometric indicators were not presented in the retrieved publications. Unpublished supplementary validation data may exist but remain inaccessible to this review, meaning our mapping only reflects publicly reported evidence rather than the full underlying psychometric profile of each instrument.

Fifth, our categorisation of theoretical grounding was extracted exclusively from original scale development papers. Descriptions classifying instruments as having complete, partial or limited theoretical foundations only reflect written records within source literature, and should not be read as independent subjective judgements of a tool’s measurement value or structural validity.

### 4.7. Recommendations for Future Research

The multiple evidence gaps identified throughout this mapping analysis establish clear priorities for subsequent psychometric and clinical research. Instead of developing additional new instruments that will further fragment an already crowded field of self-management assessment instruments for adults with colorectal cancer-related intestinal stomas, researchers are encouraged to prioritise the following work streams:

Complete full psychometric validation for existing widely adopted instruments. Formal testing of structural construct validity, criterion-related validity, cross-cultural measurement equivalence, and responsiveness should be completed for tools currently used in clinical practice. Without these supplementary data, the clinical interpretability and practical value of most scales remain unclear.

Conduct standardised cross-cultural adaptation paired with measurement invariance testing. Any scale originally developed in one cultural context and applied to distinct populations requires full adaptation following ISPOR and COSMIN guidance. Measurement invariance testing is essential to confirm consistent item interpretation across different demographic and cultural groups.

Develop and validate concise short-form versions for lengthy instruments, to lower patient response burden while retaining comprehensive coverage of core self-management domains for routine clinical screening.

Establish interpretive reference indicators for clinical use, including validated cut-off scores, minimal important difference values, and population-specific normative datasets, to support consistent score interpretation in routine ostomy care.

Explore integrated assessment models combining patient-reported questionnaires, objective clinical outcome indicators, and emerging digital stoma monitoring devices. Any novel combined assessment approach must undergo feasibility, patient acceptability, and incremental validity testing before large-scale clinical rollout.

New instrument development should only proceed if comprehensive validation and cultural adaptation of existing instruments fail to capture core self-management domains deemed vital by patients, family caregivers, and ostomy specialist nurses and surgical clinicians.

## 5. Conclusions

This scoping review systematically reviewed 12 self-management assessment instruments developed for adults with colorectal cancer-related intestinal stomas, sourced from 32 primary studies across five countries. These instruments show clear variation in dimensional construction, theoretical foundations, and the completeness of published psychometric records. While every instrument included internal consistency data, validity evidence is unevenly distributed across the field: content validity reports are relatively abundant, whereas structural validity, criterion validity, cross-cultural equivalence and responsiveness data were not identified within this set of included clinical studies. The absence of responsiveness data in the retrieved literature does not prove these instruments cannot capture long-term self-management changes; formal responsiveness testing is required prior to longitudinal outcome monitoring use. Only three instruments were constructed based on complete explicit theoretical frameworks; the majority were designed inductively from clinical experience rather than deductive theoretical derivation.

These descriptive findings demonstrate that the current evidence base supporting self-management assessment for adults with colorectal cancer-related intestinal stomas remains incomplete. Prioritised research directions should include full psychometric verification of existing tools, rigorous cross-cultural adaptation and invariance testing, and the creation of validated short-form scales where appropriate. For clinical practice, instrument selection must align with specific assessment aims and clinical environments, and all scale scores should be interpreted cautiously given the limited supporting validity evidence for most instruments.

By integrating both English and Chinese published literature, this review provides a comprehensive descriptive overview to support future cross-cultural comparative psychometric research and international collaborative measurement work. Addressing the evidence gaps outlined in this analysis will strengthen both the theoretical foundation and clinical application of self-management assessment within enterostomy nursing care for adults with colorectal cancer-related intestinal stomas.

## Data Availability

No new data were created or analyzed in this study. Data sharing is not applicable to this article.

## Supplementary Materials

The following supporting information can be downloaded at: https://www.mdpi.com/article/10.3390/healthcare14172762/s1, Supplementary S1 contains complete replicable search strategies for all ten databases; Supplementary S2 summarises dimension composition of all included assessment tools; Supplementary S3 records theoretical framework information of each instrument; Table S1 is the completed PRISMA-ScR reporting checklist.

## Abbreviations

CAESPO	Specific Self-Care for Ostomized Patients Questionnaire
EPSMQ	Enterostomy Patient Self-Management Questionnaire
EPSMS	Enterostomy Patient Self-Management Scale
ESCA	Exercise of Self-Care Agency Scale
HESSCAS	Home Enterostomy Patient Self-Care Safety Capability Assessment Scale
OSCAS	Ostomy Self-care Ability Scale
OSCI	Ostomy Self-Care Index (patient version)
CC-OSCI	Caregiver Contribution to Ostomy Self-Care Index
SSCS-EV	Stoma Self-Care Scale-early stage version
SAAQ-PRCE	Self-Care Ability Questionnaire for Patients with Permanent Rectal Cancer-Related Enterostomy
SMBQ-EP	Self-Management Behavior Questionnaire for adults with colorectal cancer-related colostomy or ileostomy
SM-CQ	Self-Management Capacity Questionnaire
CAO-EI/ESEP	Competência do Autocuidado à Ostomia de Eliminação Intestinal

## Appendix A

**Table A1:** Basic characteristics of the included studies (n = 32).

No.	Included Studies	Country	Year of Publication	Sample Size	Study Center	Research Objective	Study Type	Tool Name	Assessment Method	Outcome Indicators
1	Huang & Yan, 2025 [16]	China	2025	200	Multi-center	Tool development and validation	Cross-sectional study	Home Enterostomy Patient Self-Care Safety Capability Assessment Scale	Delphi consultation & Psychometric evaluation	Reliability and validity of the tool
2	Li, Y., et al., 2024 [17]	China	2024	422	Multi-center	Tool application	Cross-sectional study	Enterostomy Patient Self-Management Questionnaire	Patient self-report	Self-management capacity; Psychosocial adaptation; Self-efficacy; Coping style; Perceived social support
3	Wang, D., et al., 2024 [18]	China	2024	104	Single-center	Tool application	Randomized controlled trial	Exercise of Self-Care Agency Scale	Patient self-report	Anal function; self-management ability; Hope level
4	Pei et al., 2024 [19]	China	2024	203	Single-center	Tool application	Pilot study	Ostomy Self-Care Scale–Early Version	Patient self-report	Nursing measure completion rate; Patients’ self-management ability; Nurses’ satisfaction with nursing information system
5	Wang, Q., et al., 2024 [20]	China	2024	102	Single-center	Tool development and application	Randomized controlled trial	Self-Care Ability Questionnaire for Patients with Permanent Rectal Cancer-Related Enterostomy	Delphi consultation & Patient self-report	Psychosocial adaptability; Ostomy self-management ability; Incidence of ostomy complications; Quality of life
6	Ni et al., 2024 [21]	China	2024	170	Multi-center	Tool application	Cross-sectional study	Self-Management Behavior Questionnaire	Patient self-report	Scores of self-management behavior and its influencing factors
7	Li, J., & Jiao, 2024 [22]	China	2024	96	Multi-center	Tool application	Randomized controlled trial	Enterostomy Patient Self-Management Scale	Patient self-report	Ostomy stress; Self-management capacity; Quality of life
8	Wu & Zhai, 2024 [23]	China	2024	124	Single-center	Tool application	Cross-sectional study	Exercise of Self-Care Agency Scale	Patient self-report	self-management ability; Hope level; Social support level; Self-efficacy level
9	Xu et al., 2024 [24]	China	2024	179	Single-center	Tool application	Cross-sectional study	Self-Management Behavior Questionnaire	Patient self-report	Scores of self-management capacity and its influencing factors
10	Li, S., et al., 2024 [25]	China	2024	177	Single-center	Tool application	Cross-sectional study	Ostomy Self-care Ability Scale	Patient self-report	Readiness for discharge; self-management ability; Quality of life
11	An et al., 2023 [26]	China	2023	120	Single-center	Tool development and application	Cross-sectional study	Self-Management Capacity Questionnaire	Delphi consultation & Patient self-report	Self-management capacity and its influencing factors; Skin condition around ostomy
12	Zhang, Y., et al., 2023 [27]	China	2023	100	Single-center	Tool application	Randomized controlled trial	Exercise of Self-Care Agency Scale	Patient self-report	Ostomy acceptance; self-management ability; Optimism score; Pessimism score; Quality of life; Incidence of ostomy complications
13	Ge et al., 2023 [28]	China	2023	80	Single-center	Tool application	Randomized controlled trial	Exercise of Self-Care Agency Scale	Patient self-report	self-management ability; Quality of life; Incidence of ostomy-related complications
14	Iovino et al., 2023 [29]	Italy	2023	252	Multi-center	Tool application	Cross-sectional study	Ostomy Self-Care Index	Patient self-report	Self-care behavior; Caregiver contribution
15	Niu et al., 2023 [30]	China	2023	390	Single-center	Tool application	Cross-sectional study	Self-Management Behavior Questionnaire	Patient self-report	Self-management capacity; Psychological resilience; Ostomy acceptance
16	Zhang, M., et al., 2022 [31]	China	2022	93	Single-center	Tool application	Randomized controlled trial	Ostomy self-management ability Scale	Patient self-report	Self-efficacy; self-management ability; Quality of life
17	Hu et al., 2022 [32]	China	2022	115	Single-center	Tool application	Cross-sectional study	Stoma Self-Care Scale-early stage version	Patient self-report	Early self-management ability; Readiness for discharge and its influencing factors
18	Du et al., 2022 [33]	China	2022	200	Multi-center	Tool development and validation	Cross-sectional study	Self-management Behavior Questionnaire for patients with enterostomy	Delphi consultation & Psychometric evaluation	Self-management behavior; Quality of life
19	Yan et al., 2022 [34]	China	2022	74	Multi-center	Tool application	Longitudinal study	Stoma Self-care Scale-Early Stage Version	Patient self-report	Quality of life; Self-efficacy; self-management ability
20	Song et al., 2021 [35]	China	2021	85	Multi-center	Tool application	Longitudinal study	Self-Management Capacity Questionnaire	Patient self-report	Self-management capacity; Self-efficacy; Ostomy-related complications
21	Cheng et al., 2021 [36]	China	2021	195	Single-center	Tool application	Quasi-experimental study	Exercise of Self-Care Agency Scale	Patient self-report	Quality of discharge guidance; self-management ability; Incidence of ostomy-related complications
22	Collado-Boira et al., 2021 [37]	Spain	2021	139	Multi-center	Tool application	Cross-sectional study	Specific Self-Care for Ostomized Patients Questionnaire	Patient self-report	self-management ability; Health-related quality of life
23	Wang et al., 2021 [12]	China	2021	410	Multi-center	Tool application	Cross-sectional study	Exercise of Self-Care Agency Scale	Patient self-report	self-management ability; Perceived social support; Perceived stress
24	Zhang et al., 2020 [38]	China	2020	119	Multi-center	Tool application	Randomized controlled trial	Exercise of Self-Care Agency Scale	Patient self-report	Psychological resilience; self-management ability; Quality of life; Complications
25	Zhou et al., 2019 [39]	China	2019	104	Single-center	Tool application	Quasi-experimental study	Self-Management Capacity Questionnaire	Patient self-report	Incidence of ostomy complications; Ostomy self-management capacity
26	Che et al., 2019 [40]	China	2019	84	Single-center	Tool application	Randomized controlled trial	Exercise of Self-Care Agency Scale	Patient self-report	Mastery of disease knowledge; Ostomy self-management ability
27	Li, Y., et al., 2019 [41]	China	2019	120	Single-center	Tool development and application	Cross-sectional study	Enterostomy Patient Self-Management Scale	Delphi consultation & Patient self-report	Early self-management capacity and its influencing factors
28	Collado-Boira et al., 2018 [42]	Spain	2018	125	Multi-center	Tool development and validation	Longitudinal study	Specific Self-Care for Ostomized Patients Questionnaire	Delphi consultation & Psychometric evaluation	Construct validity; Internal consistency; test–retest reliability
29	Luan & Wu, 2017 [43]	China	2017	144	Multi-center	Tool development and application	Cross-sectional study	Ostomy Self-care Ability Scale	Delphi consultation & Patient self-report	self-management ability and its influencing factors
30	Silva et al. 2016 [44]	Portugal	2016	180	Single-center	Tool development	Methodological study	Competência do Autocuidado à Ostomia de Eliminação Intestinal		Knowledge; Self-monitoring; Interpretation; Decision-making; Execution; Negotiation and use of health resources
31	Liao & Qin, 2014 [45]	China	2014	76	Single-center	Tool application	Cross-sectional study	Exercise of Self-Care Agency Scale	Patient self-report	Quality of life; self-management ability; Herth Hope Index
32	Li, Q., & Zhang, 2013 [46]	China	2013	103	Multi-center	Tool application	Cross-sectional study	Ostomy Self-Care Scale–Early Version	Patient self-report	Ostomy self-management ability in early discharge period

**Table A2:** Characteristics of included assessment tools (n = 12).

Tool Name	Developer	Scoring Method	Number and Names of Dimensions	Number of Items	Reliability	Validity	Strengths	Limitations
HESSCAS	Huang F et al.	5-point Likert scale	3: Knowledge; Belief; Behavior	35	Cronbach’s α = 0.977; Dimension Cronbach’s α: 0.965, 0.930, 0.906	S-CVI = 0.971; I-CVI = 0.833–1.000	Based on KAP and self-efficacy theory; includes home care and complication monitoring subdimension	Criterion-related validity untested; Long item pool may increase participant response burden
EPSMQ	Han S	5-point Likert scale	5: Ostomy care management; Daily life management; Information management; Symptom management; Psychological management	30	Cronbach’s α = 0.916	Content validity = 0.903	Five subdomains covering physical and psychological ostomy self-management	Dependent on patient self-report; Cross-cultural applicability remains unvalidated
SAAQ-PRCE	Wang QQ et al.	5-point Likert scale	4: Not specified	31	Cronbach’s α = 0.805	I-CVI/S-CVI ranged from 0.811	Developed for adults with colorectal cancer–related permanent intestinal stomas	Dimension structure is not explicitly reported
SSCS-EV	Zhang JE et al.	5-point Likert scale	Not specified	10	Cronbach’s α = 0.9548; Split-half reliability = 0.9424	Content validity = 0.94	Concise 10-item scale for early postoperative stoma patients	Limited to early-stage ostomy populations
EPSMS	Li YM et al.	5-point Likert scale	5: Ostomy care management; Symptom care management; Daily life care management; Functional exercise management; Psychological care management	21	Cronbach’s α = 0.85	Content validity = 0.92	Covers ostomy care, symptom management and daily-life dimensions	Limited clinical application data across cohorts
ESCA	Liu YJ et al. (Chinese version)	4-point Likert scale	4: Self-care skills; Self-care responsibility; Self-care concept; Health knowledge level	43	Cronbach’s α = 0.820; test–retest reliability = 0.837;	Not specified	Widely used generic self-care instrument with four dimensions	Relatively long item pool; Dependent on self-reported responses; Psychometric properties vary across adapted versions; Validity of Chinese translations needs further verification
	Wang Laffrey (Chinese version)	5-point Likert scale			Cronbach’s α = 0.90; test–retest reliability = 0.87	Not specified		
	Kearney et al.	4/5-point Likert scale			Cronbach’s α = 0.92	Content validity = 0.98		
SM-CQ	An Q et al.	5-point Likert scale	5: Self-responsibility; Self-ostomy care skills; Daily life; Psychology; Health knowledge	30	Cronbach’s α = 0.86	Construct validity = 0.87	Covers physical, psychological and knowledge-related self-management	Validation data only available for Chinese stoma populations
OSCI	Villa et al.	5-point Likert scale	3: Self-care maintenance; Self-care monitoring; Self-care management	22	Cronbach’s α = 0.97, 0.95, 0.93	Not specified	Three subdomains derived from Riegel’s Middle-Range Self-Care Theory	Originally developed in Italy; cross-cultural validation missing
CC-OSCI	Villa et al.	5-point Likert scale	3: Caregiver contribution to maintenance, monitoring, management	22	Cronbach’s α: 0.97, 0.93, 0.91	Not specified	Parallel tool measuring caregivers’ contribution to ostomy self-care	Validity not reported
OSCAS	Gao QW; Gu NP	Self-willingness: 4-point Likert scale; Knowledge: Correct/Incorrect (1/0); Skill:Can/Cannot (1/0)	3: Self-care willingness; Self-care knowledge; Self-care skills	38	Cronbach’s α = 0.85; Dimension Cronbach’s α = 0.96, 0.82, 0.81	Content validity = 0.86	Separately assesses willingness, knowledge and operational skills	Long item pool may impose response burden; Dependent on self-report; Cross-cultural applicability unclear
CAESPO	Collado-Boira et al.	4-point Likert scale	3: General self-care; Self-care for personal development and social interaction; Specific self-care	57	Cronbach’s α = 0.889; test–retest reliability = 0.987	Structural equation modelling results from original validation: χ^2^ = 43.132, RMSEA = 0.155; CFI = 0.967; IFI = 0.968 (discrepant fit indices observed)	Includes exclusive subdomain for social participation	Long questionnaire may increase participant burden
SMBQ-EP	Du XY et al.	5-point Likert scale	5: Dietary behavior; Psychosocial behavior; Symptom management behavior; Medical adherence behavior; Information management behavior	40	Cronbach’s α = 0.972; Dimension Cronbach’s α = 0.797–0.939	I-CVI = 0.80–1.00; II-Construct validity verified by EFA, cumulative variance contribution rate = 65.42%	Behavior-focused; includes dedicated dietary and medication adherence subdomains	Long item pool; Cross-cultural applicability unvalidated
CAO-EI/ESEP	Silva et al.	4-point Likert scale	Knowledge; Self-monitoring; Interpretation; Decision-making; Execution; Negotiation and use of health resources	45	Cronbach’s α = 0.93 (discharge), 0.91 (community); all subscales α > 0.63; no test–retest reliability	Content validity verified via 5-expert focus group; no construct or criterion validity	Covers discharge and community settings; simple wording for low-literacy respondents	Reverse scoring may complicate interpretation; Test–retest and inter-rater reliability data unavailable

Note: Multiple culturally adapted revised versions of the ESCA scale are listed separately in this table to display divergent psychometric data, while ESCA is counted as one single core instrument in all quantitative frequency statistics.
