# Supplementary material for: Self-Management Assessment Instruments for Adults with Colorectal Cancer-Related Intestinal Stomas: A Scoping Review
Source: Healthcare (Basel). 2026 Sep 1;14(17):2762. doi: 10.3390/healthcare14172762 (PMC13565473; doi:10.3390/healthcare14172762)
Supplement: Supplementary file 1 [file healthcare-14-02762-s001.zip › Supplementary S1.pdf]

Supplementary S1. Search strategy

Revision Note :per Reviewer Suggestions The initial draft search contained broad nursing-related keywords. Following the reviewer's advice, all redundant nursing search terms were fully removed from each database's search syntax, and all databases were re-run to generate the updated record counts shown in the "Results" column. The total raw hits of the revised broad search = 4623. The original unoptimized retrieval volume (8937) in the first draft is discarded and no longer adopted in this revised manuscript.

Deviations from the pre-registered OSF protocol:

Two nursing-focused databases (CINAHL, Scopus) were added after protocol registration;

A dedicated instrument-targeted supplementary search was newly implemented on 31 July 2026;

All broad nursing keywords were deleted from full search strategies as requested by the reviewer, with all databases re-searched to update retrieval results.

Database	Search terms	Restrictions	Results
PubMed	**#1**: "colostomy"[MeSH Terms] OR "enterostomy"[MeSH Terms] OR "ileostomy"[MeSH Terms] OR "ostomy"[Title/Abstract] OR "stoma"[Title/Abstract] OR "intestinal stoma"[Title/Abstract] OR "colon stoma"[Title/Abstract] OR "fecal stoma"[Title/Abstract] OR "bowel stoma"[Title/Abstract] OR "ostomate"[Title/Abstract]<br><br>**#2:** "self-care"[Title/Abstract] OR "self-management"[Title/Abstract] OR "self-efficacy"[Title/Abstract] OR "self-agency"[Title/Abstract]<br><br>**#3**: "assessment"[Title/Abstract] OR "evaluation"[Title/Abstract] OR "measurement"[Title/Abstract] OR "psychometric"[Title/Abstract] OR "reliability"[Title/Abstract OR "validity"[Title/Abstract] OR "responsiveness"[Title/Abstract] OR "instrument"[Title/Abstract] OR "scale"[Title/Abstract] OR "questionnaire"[Title/Abstract] OR "inventory"[Title/Abstract] OR "index"[Title/Abstract]<br><br>**#4:**#1 AND #2 AND #3	Language: English and Chinese Published :from the database inception to 31 December 2025	**179**
Embase	('colostomy'/exp OR 'enterostomy'/exp OR 'ileostomy'/exp OR ostomy:ti,ab,kw OR stoma:ti,ab,kw OR 'intestinal stoma':ti,ab,kw OR 'colon stoma':ti,ab,kw OR 'fecal stoma':ti,ab,kw OR 'bowel stoma':ti,ab,kw OR ostomate:ti,ab,kw) AND ('self care'/exp OR 'self management'/exp OR 'self-care':ti,ab,kw OR 'self-management':ti,ab,kw OR 'self-efficacy':ti,ab,kw OR 'self-agency':ti,ab,kw) AND (assessment:ti,ab,kw OR evaluation:ti,ab,kw OR measurement:ti,ab,kw OR psychometric:ti,ab,kw OR reliability:ti,ab,kw OR validity:ti,ab,kw OR responsiveness:ti,ab,kw OR instrument:ti,ab,kw OR scale:ti,ab,kw OR questionnaire:ti,ab,kw OR inventory:ti,ab,kw OR index:ti,ab,kw)	Language: English and Chinese Published :from the database inception to 31 December 2025	**340**
Cochrane Library	**#1:** ostomy OR stoma OR intestinal stoma OR colon stoma OR fecal stoma OR bowel stoma OR colostomy OR enterostomy OR ileostomy OR ostomate<br><br>**#2:** self-care OR self-management OR self-efficacy OR self-agency<br><br>**#3:** assessment OR evaluation OR measurement OR psychometric OR reliability OR validity OR responsiveness OR instrument OR scale OR questionnaire OR inventory OR index<br><br>**#4:** #1 AND #2 AND #3	Language: English and Chinese Published :from the database inception to 31 December 2025	**184**
Web of Science	TS=((ostomy OR stoma OR "intestinal stoma" OR "colon stoma" OR "fecal stoma" OR "bowel stoma" OR colostomy OR enterostomy OR ileostomy OR ostomate) AND ("self-care" OR "self-management" OR "self-efficacy" OR "self-agency") AND (assessment OR evaluation OR measurement OR psychometric OR reliability OR validity OR responsiveness OR instrument OR scale OR questionnaire OR inventory OR index))	Language: English and Chinese Published :from the database inception to 31 December 2025	**189**

CINAHL	**S1:** (MH "Colostomy") OR (MH "Enterostomy") OR (MH "Ileostomy") OR TI (colostomy OR enterostomy OR ileostomy OR stoma OR "intestinal stoma" OR "fecal stoma" OR "bowel stoma" OR ostomate) OR AB (colostomy OR enterostomy OR ileostomy OR stoma OR "intestinal stoma" OR "fecal stoma" OR "bowel stoma" OR ostomate)<br><br>**S2:** (MH "Self Care") OR (MH "Self-Management") OR TI ("self-care" OR "self-management" OR "self-efficacy" OR "self-agency") OR AB ("self-care" OR "self-management" OR "self-efficacy" OR "self-agency")<br><br>**S3:** (MH "Psychometrics") OR (MH "Questionnaires") OR TI (assessment OR evaluation OR measurement OR scale OR questionnaire OR index OR inventory OR psychometric* OR reliability OR validity OR responsiveness) OR AB (assessment OR evaluation OR measurement OR scale OR questionnaire OR index OR inventory OR psychometric* OR reliability OR validity OR responsiveness)<br><br>**S4:** S1 AND S2 AND S3	Language: English and Chinese Published :from the database inception to 31 December 2025	**240**
Scopus	TITLE-ABS-KEY (colostomy OR enterostomy OR ileostomy OR stoma OR "intestinal stoma" OR "fecal stoma" OR "bowel stoma" OR ostomate) AND TITLE-ABS-KEY ("self-care" OR "self-management" OR "self-efficacy" OR "self-agency") AND TITLE-ABS-KEY (assessment OR evaluation OR measurement OR scale OR questionnaire OR index OR inventory OR psychometric* OR reliability OR validity OR responsiveness OR "cross-cultural adaptation") AND (LIMIT-TO (LANGUAGE , "English") OR LIMIT-TO (LANGUAGE , "Chinese")) AND PUBYEAR < 2026	Language: English and Chinese Published :from the database inception to 31 December 2025	**346**
SinoMed	("人工肛门"[常用字段:智能] OR "回肠造口"[常用字段:智能] OR "结肠造口"[常用字段:智能] OR "肠造口"[常用字段:智能] OR "造口"[常用字段:智能] OR "造瘘"[常用字段:智能] OR "肠造瘘"[常用字段:智能]) AND("自我照护"[常用字段:智能] OR "自我管理"[常用字段:智能] OR "自我护理"[常用字段:智能] OR "自我效能"[常用字段:智能]) AND("评估"[常用字段:智能] OR "评价"[常用字段:智能] OR "测量"[常用字段:智能] OR "识别"[常用字段:智能] OR "筛查"[常用字段:智能] OR "工具"[常用字段:智能] OR "量表"[常用字段:智能] OR "指标"[常用字段:智能] OR "问卷"[常用字段:智能])	Language: Chinese Published :from the database inception to 31 December 2025	**1181**
CNKI	主题=(((人工肛门 OR 回肠造口 OR 结肠造口 OR 肠造口 OR 造口 OR 造瘘 OR 肠造瘘) AND (自我照护 OR 自我管理 OR 自我护理 OR 自我效能) AND (评估 OR 评价 OR 测量 OR 识别 OR 筛查 OR 工具 OR 量表 OR 指标 OR 问卷)))	Language: Chinese Published :from the database inception to 31 December 2025	**970**
Wangfang Data	(主题:(人工肛门 OR 回肠造口 OR 结肠造口 OR 肠造口 OR 造口 OR 造瘘 OR 肠造瘘) and 题名或关键词:(自我照护 OR 自我管理 OR 自我护理 OR 自我效能) and 题名或关键词:(评估 OR 评价 OR 测量 OR 识别 OR 筛查 OR 工具 OR 量表 OR 指标 OR 问卷）	Language: Chinese Published :from the database inception to 31 December 2025	**948**
VIP Chinese Journal Database	((((((((题名或关键词=人工肛门 OR 题名或关键词=回肠造口) OR 题名或关键词=结肠造口) OR 题名或关键词=肠造口) OR 题名或关键词=造口) OR 题名或关键词=造瘘) OR 题名或关键词=肠造瘘) AND (((题名或关键词=自我照护 OR 题名或关键词=自我管理) OR 题名或关键词=自我护理) OR 题名或关键词=自我效能)) AND ((((((((题名或关键词=评估 OR 题名或关键词=评价) OR 题名或关键词=测量) OR 题名或关键词=识别) OR 题名或关键词=筛查) OR 题名或关键词=工具) OR 题名或关键词=量表) OR 题名或关键词=指标) OR 题名或关键词=问卷))	Language: Chinese Published :from the database inception to 31 December 2025	**46**

Supplementary Instrument-Targeted Search Strategy: This targeted search was conducted across all 10 databases on 31 July 2026 after the revised broad search (without nursing keywords). Full official names, abbreviations and developer names of the 12 instruments were used as supplementary search terms to capture missing scale development and validation papers. No additional eligible unique articles were retrieved via this targeted search. List of retrieval instruments: ESCA, EPSMQ, OSCAS, SSCS-EV, CAESPO, EPSMS, SAAQ-PRCE, OSCI, HESSCAS, CAO-EI/ESEP, SMBQ-EP,SM-CQ
