# Supplementary material for: Self-Management Assessment Instruments for Adults with Colorectal Cancer-Related Intestinal Stomas: A Scoping Review
Source: Healthcare (Basel). 2026 Sep 1;14(17):2762. doi: 10.3390/healthcare14172762 (PMC13565473; doi:10.3390/healthcare14172762)
Supplement: Supplementary file 1 [file healthcare-14-02762-s001.zip › Supplementary S2.pdf]

Supplementary S2. Frequency of Measurement Dimensions Identified Across the 12 Colorectal Cancer-Related Intestinal Stoma Self-Management Assessment Instruments

Preceding Methodological Note: Two independent researchers inductively merged semantically identical subscale dimensions extracted from Table A2. All divergent labels describing the same rehabilitation construct were grouped into unified core categories. Disagreements on dimension classification were resolved via discussion with a third senior reviewer.

Statistical counting rule: Each unique measurement instrument was counted only once; multiple revised/translated ESCA variants were not double-counted for frequency statistics. All dimension grouping procedures followed the thematic synthesis workflow described in Section 2.5 of the main manuscript. Four overarching core domains summarised in the main text were derived from the fine-grained subcategories listed below.

Overarching Core Domain (Main Text Standard Term)	Fine-Grained Sub-Dimension Labels Extracted from Scales	Number of Unique Instruments Covered	Corresponding Assessment Tools
1. Stoma Technical Care & Symptom Management(Primary physical rehabilitation domain)	Ostomy care management, self-ostomy care skills, specific ostomy self-care, symptom monitoring, symptom care management, symptom management behavior	7	EPSMQ, EPSMS, SM-CQ, CAESPO, OSCAS, SMBQ-EP, OSCI
2. Psychological Adaptation (Psychosocial core domain)	Psychological management, psychological care, psychosocial behavior, emotional adjustment	4	EPSMQ, EPSMS, SM-CQ, SMBQ-EP
3. Family & Social Interaction	Social participation, social interaction for ostomy patients	1	CAESPO
4. Health Knowledge & Self-Responsibility	Health knowledge, self-care knowledge, self-care responsibility, self-management awareness	4	ESCA, SM-CQ, OSCAS, SMBQ-EP

Supplementary Fine-Grained Single-Item Dimensions (Appeared in only one instrument each):

1. Dietary behavior (SMBQ-EP)
2. Medical adherence behavior (SMBQ-EP)
3. Functional exercise assessment (EPSMS)
4. Self-care willingness (OSCAS)
5. Self-care conceptual cognition (ESCA)
6. Self-care monitoring (OSCI)

Supplementary Note

1. Frequency definition: The “Number of Unique Instruments Covered” column counts distinct core tools (n=12), excluding duplicated Chinese adapted versions of ESCA, consistent with the quantitative analysis standard in the main text.

2. Data alignment with Discussion Section 4.1: Stoma technical and symptom-related dimensions are widely covered across most instruments, while the family-social interaction domain is only included in CAESPO, confirming the uneven dimensional coverage highlighted in the manuscript.
3. Terminology unification rule: The term self-management is used exclusively for colorectal cancer-related ostomy-specific constructs; self-care agency only refers to the generic ESCA framework, consistent with the conceptual distinction in Section 1 of the main text.
4. Abbreviation standard: All tool abbreviations strictly follow the abbreviation list in the main text.
