# Supplementary material for: Self-Management Assessment Instruments for Adults with Colorectal Cancer-Related Intestinal Stomas: A Scoping Review
Source: Healthcare (Basel). 2026 Sep 1;14(17):2762. doi: 10.3390/healthcare14172762 (PMC13565473; doi:10.3390/healthcare14172762)
Supplement: Supplementary file 1 [file healthcare-14-02762-s001.zip › Supplementary S3.pdf]

Supplementary S3.Theoretical Foundations of Included Instruments

Tool Name	Declared Theoretical Model	Dimension-Theory Matching Example	Notes
ESCA	Orem’s Self-Care Deficit Theory	“Self-care skills” corresponds to “self-care agency”	Explicit theoretical guidance was adopted during scale construction, with one-to-one correspondence between theoretical constructs and scale dimensions reported in original validation papers
HESSCAS	Knowledge-Attitude-Practice (KAP) Theory combined with self-efficacy theory	Knowledge dimension corresponds to “cognitive basis”;<br>belief dimension corresponds to “attitudinal motivation”;<br>behavior dimension corresponds to “behavioral implementation”	Clear theoretical framework guided item development
OSCI	Riegel’s Middle-Range Theory of Self-Care of Chronic Illness	Self-care maintenance, self-care monitoring and self-care management dimensions were directly derived from the core constructs of this theory	Dimensions are conceptually consistent with three core components of the theory, although formal empirical verification of theory-dimension alignment was not fully reported in the original paper

Note: This appendix records theoretical information reported in the original scale development publications of instruments that declared a theoretical basis. No subjective evaluation or grading of theoretical fitness was performed.
